# 

**Published:** 1916-12

**Authors:** 


### The Library of the Bristol Medico-Chirurgical Society.

### NINETY-FIFTH LIST OF BOOKS.

*The following books have been received since the publication of the List in September,* 1914.

*September,* 1915.

| | | | |
|---|---|---|---|
| Albutt, C. | *Diseases of the Arteries* | | 1915 |
| Ballantyne, J. W. | *Encyclopædia Medica* | Vol. I. 2nd Ed. | 1915 |
| Ballantyne, J. W. | *Encyclopædia Medica* | Vol. II. 2nd Ed. | 1915 |
| Baylisis, W. A. | *Principles of General Physiology* | | 1915 |
| Berkeley, C. and Bonney, V. | *Guide to Gynæcology* | | 1915 |
| Brown, W. and Murphy, J. | *Practitioner Encyclopædia of Medical Treatment* | | 1915 |
| Buchanan, Prof. | *Forensic Medicine and Toxicology* | | 1915 |
| Bulkley, L. D. | *Cancer and its Treatment* | | 1915 |
| Bruce and Dilling | *Materia Medica and Therapeutics* | 10th Ed. | 1915 |
| Bryce, T. H. | *Quain's Anatomy* | Vol. IV., Pt. I. 11th Ed. | 1915 |
| Chambers, R. | *Dictionary of Eminent Scotsmen* | 7 Vols. | 1868 |
| Delorme, E. | *War Surgery* | | 1915 |
| Dupuy, G. M. | *The Stretcher Bearer* | | 1915 |
| Garrison, T. H. | *A Memoir of J. S. Billing* | | 1915 |
| Glaister, G. | *Medical Jurisprudence and Toxicology* | 2nd Ed. | 1912 |
| Glaister, G. | *Medical Jurisprudence and Toxicology* | 3rd Ed. | 1915 |
| Groves, E. W. Hey | *Gunshot Injuries to Bones* | | 1915 |
| Harris, W. | *Nerve Injuries of Bones* | | 1915 |
| Hodgson, D. | *Prostrate Gland* | | 1856 |
| Hornsley, J. A. and Schmidt, R. E. | *The Modern Hospital* | | 1913 |
| Hope, E. W. | *Text-book of Public Health* | 8th Ed. | 1915 |
| Horder, T. J. | *Cerebro-Spinal Fever* | | 1915 |
| Horsley, Sir V. and Sturge, M. D. | *Alcohol and the Human Body* | 5th Ed. | 1915 |
| Hutchinson, R. and Sherrin, J. | *Index of Treatment* | 7th Ed. | 1915 |
| Jones, R. | *Injuries to Joints* | | 1915 |
| Kenwoods, H. R. | *Health in Camp* | | 1915 |
| Lejars, F. | *Urgent Surgery.* (Translated from the French by W. S. Dickie) | Vol. II. 3rd Ed. | 1915 |
| Llewellyn, Ll. J. and Jones, A. B. | *Fibrositis* | | 1915 |
| Luff, A. P. and Candy, H. C. H. | *Manual of Chemistry* | 5th Ed. | 1915 |
| Martindale, W. H. and Westcott, W. (Rev.). | *The Extra Pharmacopœia* | Vols. I. and II. 16th Ed. | 1915 |
| Mercier, C. A. | *Leper Houses and Mediæval Hospitals* | | 1915 |
| Morrison and Richardson | *Abdominal Injuries* | | 1915 |
| Morrison, R. G. | *Life a Poem* | | 1915 |

| | | |
|---|---|---|
| **Mowat, H.** .. .. | *X-rays and How to Produce and Use Them* .. | 1915 |
| **Murphy, J. K.** .. | *Wounds of the Thorax* .. .. .. .. .. .. | 1915 |
| **Nash, J. T. C.** .. | *Evolution and Disease* .. .. .. .. .. .. | 1915 |
| **Paterson, A. M.** .. | *Manual of Embryology* .. .. .. .. .. .. | 1915 |
| **Phillips, L. P.** .. | *Amaibiasis and the Dysenteries* .. .. .. | 1915 |
| **Power, D.** .. .. | *Wounds in War* .. .. .. .. .. .. .. .. | 1915 |
| **Rawlings** .. .. | *Surgery of the Head* .. .. .. .. .. .. .. | 1915 |
| **Rion, H.** .. .. | *Childbirth in Twilight Sleep* .. .. .. .. .. | 1915 |
| **Saunby, R.** .. .. | *Urgent Symptoms in Medical Practice* .. .. | 1915 |
| **Shipley, A. E.** .. | *The Minor Horror of War* .. .. .. .. .. | 1915 |
| **Short, A. R.** .. .. | *Index of Prognosis.* By various writers. Edited by A. R. Short .. .. .. .. .. | 1915 |
| **Squire, J. E.** .. .. | *Medical Hints* .. .. .. .. .. .. .. .. .. | 1915 |
| **Still, F. G.** .. .. | *Disorders and Diseases of Children* 3rd Ed. | 1915 |
| **Todd, T. W.** .. .. | *Clinical Anatomy of the Gastro-Intestinal Tract* | 1915 |
| **Thomson, H. C.** .. | *Diseases of the Nervous System* .. 11th Ed. | 1915 |
| **Thomson and Myles** | *Manual of Surgery* .. .. Vol. I. 5th Ed. | 1915 |
| **Thomson and Myles** | *Manual of Surgery* .. .. Vol. II. 5th Ed. | 1915 |
| **Urquhart, J.** .. .. | *The Life and Teaching of W. H. Gillespie* .. | 1915 |
| **Vallow, H.** .. .. | *The Inevitable Compliment* .. .. .. .. .. | 1915 |
| **Werner, L.** .. .. | *Swanzy's Diseases of the Eye* .. .. 2nd Ed. | 1915 |
| **Wright, Sir A. E.** .. | *Wound Infections* .. .. .. .. .. .. .. .. | 1915 |

### TRANSACTIONS, REPORTS, JOURNALS, etc.

| | |
|---|---|
| American Association of Genito-Urinary Surgery .. .. Vol. IX. | 1915 |
| American Association of Obstetricians, Transactions of the Vol. XXVII. | 1914 |
| American Laryngological Association, Transactions of the .. .. | 1915 |
| American Pediatric Society, Transactions of the .. Vol. XXVI. | 1914 |
| American Surgical Association, Transactions of the .. Vol. XXXII. | 1914 |
| Botany, Part II. .. .. .. .. .. .. .. .. .. .. .. .. .. .. .. | 1856 |
| College of Physicians of Philadelphia, Transactions of the Vol. XXXVI. | 1915 |
| Edinburgh Obstetric Society, Transactions of the .. Vol. XXXIX. | 1914 |
| Edinburgh Pharmacopœia .. .. .. .. .. .. .. .. .. .. .. .. | 1817 |
| Index Catalogue of the Surgeon-General of United States Army Vol. XXXIII. | 1914 |
| Journal of Laryngology .. .. .. .. .. .. .. .. .. .. .. .. .. | 1914 |
| London Dermatology Society, Transactions of the .. .. .. .. | 1914 |
| Medical Annual .. .. .. .. .. .. .. .. .. .. .. .. .. .. | 1915 |
| Medical Society of London, Transactions of the Vol. XXXVII. | 1914 |
| Medical Society of London, Transactions of the Vol. XXXVIII. | 1915 |
| Smithsonian Report, The .. .. .. .. .. .. .. .. .. .. .. | 1914 |
| Tuberculosis, Yearly Book .. .. .. .. .. .. .. Vol. I. | 1913–14 |

*The following periodicals were discontinued at the end of* 1914 :—

American Journal of the Medical Science.
Annals de Dermatologie.
Archives of the X-rays.
Archiv für klinische Chirurgie.
Bookseller, The.
British Journal of Children Diseases.
Bulletin de la Societe de Dermatologie.
Current Literature.
Duetsche Zeitschrift für Chirurgie.
Journal of Hygiene.
Journal of Obstetrics and Gynecology.
Journal of Nervous and Mental Diseases.
Journal of the Royal Sanitary Institute.
Library, The.
Library Association Record.
Library World, The.
Medical Library Association Journal.
Progressive Medicine.
Parisitology.
Revue de Chirurgie.
Surgery, Gynecology and Obstetrics.
Zeitschrift für Urologie.
Zentralblatt für Chirurgie.
Zentralblatt für Gynäkologie.
Zentralblatt für innere Medicine.

The Quarterly Journal of Medicine and Surgery, Gynecology and Obstetrics, is kindly given by two members.

### NINETY-SIXTH LIST OF BOOKS.

---

*The following have been added to the Library since September,* 1915 :—

### BOOKS.

*November,* 1916.

| | | | |
|---|---|---|---|
| **Barr, W. I. K.** .. | *Theraphy in Pulmonary Tuberculosis* .. .. | | 1916 |
| **Basu, B. D.** .. .. | *Dietitic Treatment of Diabetes* .. | 7th Ed. | 1916 |
| **Batten, F. E.** .. .. | *Acute Poliomyclitis* .. .. .. .. .. .. | | 1916 |
| **Beesley and Johnston** | *Manual of Surgical Anatomy* .. .. .. .. | | 1916 |

| | | | |
|---|---|---|---|
| **Bosanquet and Eyre** | *Serums, Vaccines, and Toxins* | 3rd Ed. | 1916 |
| **Brunton, Sir T. Lauder** | *Circulation and Respiration.* 2nd series | | 1916 |
| **Bumstead and Taylor** | *Venereal Diseases* | | 1879 |
| **Clarke, J. J.** | *Protozoa and Disease* | Pt. IV. | 1915 |
| **Coupland, S.** | *The Harveian Oration* | | 1915 |
| **Collie, T.** | *Collect Reprints.* 2nd series | | 1916 |
| **Da Costa** | *Medical Diagnosis* | | 1879 |
| **Despard, L. L.** | *Hand-Book of Massage for Beginners* | | 1916 |
| **Dockrell, N.** | *Atlas of Dermatology* | | 1905 |
| **Draper, J. W.** | *Studies in Surgical Pathological Physiology* | | 1915 |
| **Flint, F.** | *Practice of Medicine* | | 1887 |
| **Gee, S. J.** | *Medical Lectures and Clinical Aphorisms* | 4th Ed. | 1915 |
| **Greene, Clayton** | *Pye's Surgical Handicraft* | 7th Ed. | 1916 |
| **Groves, E. W. Hey** | *Modern Methods of Treating Fractures* | | 1916 |
| **Harrison, J.** | *Three Ballads* | | 1869 |
| **Hewer, J. L.** | *Our Baby* | 15th Ed. | 1916 |
| **Hutchinson and Rainy** | *Clinical Methods* | 6th Ed. | 1916 |
| **Jack, W. R.** | *Wheeler's Handbook of Medicine* | 5th Ed. | 1916 |
| **Kelson, W. H.** | *Diseases of the Throat, Nose and Ear* | | 1916 |
| **Kendrick** | *Back Injuries* | | 1916 |
| **Kettle, E. H.** | *Pathology of Tumours* | | 1916 |
| **Lancereaux** | *Treatise of Syphilis* | Vol. I. | 1868 |
| **Lancereaux** | *Treatise of Syphilis* | Vol. II. | 1869 |
| **Lane, Sir A.** | *Hare Lip and Cleft Palate* | 3rd Ed. | 1916 |
| **Mackenzie, Sir James** | *Diagnosis and Treatment in Heart Affections* | | 1916 |
| **Morrison, Rutherford** | *Surgical Contributions* | Vol. I. | 1916 |
| **Morrison, Rutherford** | *Surgical Contributions* | Vol. II. | 1916 |
| **Moullin, C. M.** | *The Biology of Tumours* | | 1916 |
| **Oliver, G.** | *Studies in Blood-Pressure* | 3rd Ed. | 1916 |
| **Penhallow, D. G.** | *Military Surgery* | | 1916 |
| **Porter, W.** | *Diseases of the Nose, Throat and Ears* (Revised by H. Evans during W. Porter's absence on Military Service) | 2nd Ed. | 1916 |
| **Ramsay, A. B.** | *Injuries of the Eyes, Nose and Throat* | | 1916 |
| **Scott** | *Gould's Practitioners Medical Dictionary* | 3rd Ed. | 1916 |
| **Scott, T. B.** | *Modern Medicine and Modern Remedies* | | 1916 |
| **Short, A. Rendle** | *When to Advise Operation in Surgical Practice* | | 1916 |
| **Sutherland, H. G.** | *Pulmonary Tuberculosis* | | 1916 |
| **Thomson, St. Clair** | *Diseases of the Nose and Throat* | 2nd Ed. | 1916 |
| **Walker, Norman** | *Introduction to Dermatology* | 6th Ed. | 1916 |
| **Warwick and Tunstall** | *First Aid to the Injured and Sick* | 8th Ed. | 1915 |
| **Warwick and Tunstall** | *Questions on First Aid to the Injured and Sick* | | 1915 |
| **Weber, T. P.** | *Traumatic Pneumonia and Tuberculosis* | | 1916 |

### JOURNALS, REPORTS, AND TRANSACTIONS, etc.

| | | |
|---|---|---|
| American Association of Obstetricians and Gynecologists, Transactions of the | Vol. XXVII. | 1915 |
| American Association of Obstetricians and Gynecologists, Transactions of the | Vol. XXVIII. | 1916 |
| American Journal of Medical Sciences | Vol. CXLVIII. | 1914 |
| American Journal of Obstetrics | Vol. LXXV. | 1914 |
| American Journal of Obstetrics | Vol. LXXVI. | 1915 |
| American Journal of Surgery | Vol. XXVIII. | 1914 |
| American Laryngological Association's Report | | 1915 |
| American Larnygology, Rhinology, Otology Society, Transactions of the | | 1915 |
| American Ophthalmology Society, Transactions of the | Vol. XIV., Pt. III. | 1915 |
| American Ophthalmology Society, Transactions of the | Vol. XV. Pt. I. | 1916 |
| American Pediatrics Society, Transactions of the | | 1915 |
| American Surgical Association, Transactions of the | Vol. XXXIII. | 1915 |
| American Urological Association, Transactions of the | Vol. VIII. | 1914 |
| American Urological Association, Transactions of the | Vol. IX. | 1915 |
| Annual Report of the Smithsonian Institute | | 1915 |
| Bellevue and Allied Hospitals, Report of the | | 1914 |
| Boston Medical and Surgical Journal | Vol. CLXXI. | 1914 |
| Boston Medical and Surgical Journal | Vol. CLXXII. | 1915 |
| Bristol Medico-Chirurgical Society | Vol. XXXII. | 1914 |
| British Journal of Children's Diseases | Vol. XI. | 1914 |
| British Journal of Dermatology | Vol. XXVI. | 1914 |
| British Medical Journal | Vol. II. | 1914 |
| British Medical Journal | Vol. I. | 1915 |
| Canadian Medical Journal | Vol. IV. | 1914 |
| Clinical Journal | Vol. XLIII. | 1914 |
| Cyclopædia of English Literature | Vol. I. | 1858 |
| Cyclopædia of English Literature | Vol. II. | 1860 |
| Edinburgh Medical Journal | Vol. XIII. | 1914 |
| Edinburgh Medical Journal | Vo. XIV. | 1915 |
| Edinburgh University, Calendar of the | | 1916–7 |
| Encyclopædia Medica | Vol. III. 2nd Ed. | 1916 |
| Glasgow Medical Journal | Vol. LXXXII. | 1914 |
| Glasgow Medical Journal | Vol. LXXXIII. | 1915 |
| Guy's Hospital Reports | Vol. LIII. | 1914 |
| Index Catalogue of the Library of the Surgeon-General of the United States Army | | 1915 |
| Indian Medical Gazette | Vol. XLIX. | 1914 |
| International Abstract of Surgery | Vol. XIX. | 1914 |
| International Abstract of Surgery | Vol. XX. | 1915 |
| John Hopkins Bulletin | Vol. XXV. | 1914 |
| Journal of American Medical Association | Vol. LXIII. | 1914 |
| Journal of Hygiene | Vol. XIV. | 1914 |

| | | |
|---|---|---|
| Journal of Laryngology | Vol. XXIX. | 1914 |
| Journal of Medical Research | Vol. XXXI. | 1914 |
| Journal of Medical Research | Vol. XXXII. | 1914–5 |
| Journal of Medical Research | Vol. XXXIII. | 1915 |
| Journal of Mental Science | Vol. LX. | 1914 |
| Journal of Nervous and Mental Diseases | Vol. LXI. | 1914 |
| Journal of Obstetrics and Gynecology | Vol. XXV. | 1914 |
| Journal of Obstetrics and Gynecology | Vol. XXVI. | 1915 |
| Journal of Parasitology | Vol. VII. | 1914 |
| Journal of the Royal Sanitary Institute | Vol. XXXV. | 1914 |
| Journal of Tropical Medicine | Vol. XVII. | 1914 |
| Journal of Tuberculosis | Vol. VIII. | 1914 |
| Lancet, The | Vol. II. | 1914 |
| Lancet, The | Vol. I. | 1915 |
| Medical Annual, The | | 1916 |
| Medical Annual Index, The | | 1915 |
| Medical Press and Circular, The | Vol. CL. | 1914 |
| Medical Press and Circular, The | Vol. CLI. | 1915 |
| Medical Register, The | Vol. I. | 1825 |
| Medical Review, The | Vol. XVII. | 1914 |
| Medical Review, The | Vol. I. | 1841 |
| Medical Review, The | Vol. II. | 1842 |
| Medical Society's Transactions, The | Vol. XXXVII. | 1916 |
| Medical and Surgical Report of the Roosevelt Hospital | | 1915 |
| Mercy and Truth | Vol. II. | 1898 |
| New Zealand Medical Directory, The | | 1915 |
| Ophthalmology Society of the United Kingdom, Transactions of the | | 1915 |
| Pharmaceutical Society of Great Britain, Calendar of the | | 1915–6 |
| Practitioner, The | Vol. XCIII. | 1914 |
| Practitioner, The | Vol. XCIV. | 1915 |
| Proceedings of the Royal Society of Medicine | Vol. VII. | 1914 |
| Public Health | Vol. XXVII. | 1914 |
| Public Health | Vol. XXVIII. | 1915 |
| Medical Officer, The | Vol. XII. | 1914 |
| Medical Officer, The | Vol. XIII. | 1915 |
| Royal Academy of Medicine of Ireland, Transactions of the | Vol. XXXIII. | 1915 |
| Royal Academy of Medicine of Ireland, Transactions of the | Vol. XXXIV. | 1916 |
| Royal College of Surgeons of England, Calendar of the | | 1916–7 |
| Rules for the Prevention of Infectious Diseases in Schools | | 1915 |
| St. Bartholomew's Hospital Reports | | 1915 |
| St. John Hopkins Hospital Reports | | 1916 |
| St. Thomas Hospital Reports | | 1913 |
| Surgery, Gynecology and Obstetrics | Vol. XIX. | 1914 |
| Surgery, Gynecology and Obstetrics | Vol. XX. | 1915 |
| Therapeutic Gazette | Vol. XXXVIII. | 1914 |
| West London Medical Journal | Vol. XIX. | 1914 |
| Year-Book of the Open Air Schools and Children's Sanatoriums | | 1915 |

### Local Medical Notes.

Results of recent examinations :—

UNIVERSITY OF BRISTOL.—*M.B., Ch.B.* (*Final*) *:* O. C. M. Davis,* Hilda H. Ewens.* *Part I. :* Edwin J. Ball. *Intermediate :* Elizabeth Casson, B. A. Astley Weston, R. F. White. *Second Examination, Part I. :* Hilda M. Brown, S. Datta, Marjorie S. Neville, T. H. A. Pinniger. *First Examination :* W. L. C. Cossham, S. Datta, H. F. Parsons, Nan Susan J. Roberts, Marjorie Wasworth.

L.D.S.—*Final Examination :* R. L. O'Sullivan. *Second Examination :* J. L. Delicati, W. H. Shipway. *First Examination :* N. Bayly, S. W. Bodenham, L. O. Bodey, V. Bourgeois. *Examination for D.P.H.* (*Part I.*) *:* J. H. Harper.

UNIVERSITY OF LONDON.—*Third Examination for M.B., B.S. :* D. G. C. Tasker.* *Group II. :* A. W. Adams,* *Second Examination for Anat. Physiology :* R. F. White.

**Royal College of Physicians and Surgeons.**—CONJOINT L.R.C.P., M.R.C.S. : Louisa Margaret Lister,* A. G. Morris,* D. M. Smith,* D. G. C. Tasker,* G. C. Williams,* H. B. Logan. *Medicine :* F. C. A. Frith, D. L. Lees, H. Archer. *Surgery :* D. L. Lees, R. H. Tasker, T. D. Morgan, Emmerson Healin. *Midwifery :* A. H. Moris. *Anatomy and Physiology :* N. C. Cooper. *Chemistry and Physics :* R. M. Ealand. *Biology :* A. E. Mills.

**Royal College of Surgeons.**—*Primary, Anatomy and Physiology :* G. V. W. Anderson. *L.D.S.* (*Final*), *Part I. :* A. Williams. *Preliminary L.D.S. :* E. R. Pfaff.

**Society of Apothecaries of London.**—L.S.A. : H. Archer.* *Anatomy and Physiology :* J. F. E. Burns, W. H. Hazell, A. E. Young. *Physiology :* N. H. Kettlewood.

* Denotes qualification.

### INDEX.

Ackland, Major W. R.—The effect of general conditions on the quality of the teeth, 118.

Blood-stains in the Tropics, Forensic Medicine of—F. Shingleton Smith, 82.

**Books, Reviews of—**

Abdominal injuries—Prof. Rutherford Morrison, 97.
Allbutt, Sir C.—Diseases of the arteries, including angina pectoris, 180.
Arteries, Diseases of the, including angina pectoris—Sir C. Allbutt, 180.
Blood-pressure, physiological and clinical, Studies in—Dr. G. Oliver, 181.
Brunton, Sir T. Lauder—Circulation and respiration, 185.
Catalogue (Index) of the Library of the Surgeon-General's Office of the United States army, 99.
Cerebro-spinal fever—T. J. Horder, 97.
Circulation and respiration—Sir T. Lauder Brunton, 185.
Dermatology, Introduction to—Dr. Norman Walker, 183.
Encyclopædia Medica, vol. III., 98.
Fractures, On modern methods of treating —E. W. Hey Groves, 182.
Genito urinary organs, Surgical diseases and injuries of the—J. W. Thomson Walker, 96.
Groves, E. W. Hey—On modern methods of treating fractures, 182.
Groves, E. W. Hey—Gunshot injuries of bones, 97.
Gunshot injuries of bones—E. W. Hey Groves, 97.
Harris, W.—Nerve injuries and shock, 97.
Heart affections, Principles of diagnosis and treatment in—Sir James Mackenzie, 184.
Horder, T. J.—Cerebro-spinal fever, 97.
Injuries of the eyes, nose, throat and ears—Major A. Maitland Ramsay, Major D. Grant, Capt. L. Whale, and Capt. C. E. West, 97.
Joints, Injuries to—Major R. Jones, 97.
Jones, Major R.—Injuries to joints, 97.
Mackenzie, Sir James—Principles of diagnosis and treatment in heart affections, 184.
Medicine and some modern remedies, Modern—T. B. Scott, 98.

**Books, Reviews of** (*continued*)—

Medical Annual, 1916, The, 99.
Medical hints—Col. J. Edward Squire, 97.
Morrison, Prof. Rutherford—Abdominal injuries, 97.
Murphy, J. Keogh—Wounds of the thorax in war, 97.
Nerve injuries and shock—W. Harris, 97.
Nose and throat, Diseases of the—Sir St. Clair Thomson, 184.
Oliver, Dr. G.—Studies in blood-pressure, physiological and clinical, 181.
Operation in general practice, When to advise—A. Rendle Short, 182.
Oxford war primers, 96.
Porter, Dr. W. G.—Diseases of the throat, nose and ear, 184.
Power, Lieut.-Col. D'Arcy—Wounds in war, 96.
Ramsay, Major F. Maitland, Major D. Grant, Capt. L. Whale, and Capt. E. C. West—Injuries to the eyes, nose, throat and ears, 97.
Rawling, Major R.—Surgery of the head, 97.
Scott, T. B.—Modern medicine and some modern remedies, 98.
Short, A. Rendle—When to advise operation in general practice, 182.
Squire, Col. J. E.—Medical hints, 97.
Surgery of the head—Major R. Rawling, 97.
Thomson, Sir St. Clair—Diseases of the nose and throat, 184.
Throat, nose and ear, Diseases of the—Dr. W. G. Porter, 184.
Walker, J. W. Thomson—Surgical diseases and injuries of the genito-urinary organs, 96.
Walker, Dr. Norman—Introduction to Dermatology, 183.
Wound infections—Sir A. E. Wright, 93.
Wounds in war—Lieut.-Col. D'Arcy Power, 96.
Wounds of the thorax in war—J. Keogh Murphy, 97.
Wright, Sir A. E.—Wound infections, 93.

Bristol Medico-Chirurgical Society, Meetings of the, 100.
Bristol Medico-Chirurgical Society, The Library of, 195.
British surgery in France, Some aspects of—Dr. James Swain, 128

Clarke, Lieut.-Col. J. Michell—Some neuroses of the war, 49.
Cotton, Captain W.—Ten months in France with a field service X-ray outfit, 144.
Coombs, Capt. F. Carey—Medicine and surgery in Mesopotamia, 136.

Death of Sir George White, Bart., President of the Bristol Royal Infirmary, 194.
Dermatology, Report on—Dr. H. Waldo, 178.

Ear, nose and throat specialist at one of the bases, Experiences of an—Capt. J. P. I. Harty, 39.
Editorial notes, 100, 186.
Edgeworth, Dr. F. H.—The art of medicine in the age of Homer, 105.
Egypt, On the work of a military general hospital in—E. H.Groves, 11.
Enteric group of diseases as seen in an isolation hospital in France, Notes on the—Capt. J. M. Fortescue-Brickdale, 72.
Ewens, John—Obituary notice of, 201.

Forensic medicine of blood-stains in the Tropics—F. Shingleton Smith, 82.
Fortescue-Brickdale, Capt. J. M.—Notes on the enteric group of diseases as seen in an isolation hospital in France, 72.
France, Some aspects of British surgery in—Dr. James Swain, 128.
France with a field service X-ray outfit, Ten months in—Capt. W. Cotton, 144.

Gordon, W.—The reduction of disabilities from wounds in war, 1.
Grace, Alfred—Obituary notice of, 102.
Grace, Dr. W. G.—Obituary notice of, 103.
Groves, E. Hey—On the work of a military general hospital in Egypt, 11.

Hartnell, Capt. E. B.—Obituary notice of, 101.
Harty, Capt. J. P. I.—Experiences of an ear, nose and throat specialist at one of the bases, 39.
Horsley, The late Sir Victor—A personal note on, 186.

Library of the Bristol Medico-Chirurgical Society, 195.
Local medical notes, 202.

Medicine and surgery in Mesopotamia—Capt. Carey F. Coombs, 136.
Medicine in the age of Homer, The art of—Dr. F. H. Edgeworth, 105.
Meetings of Societies, 100.
Meningococcal and other infections, Note on the technique of sphenoidal sinus exploration for—Major P. Watson-Williams, 21.
Mesopotamia, Medicine and surgery in—Capt. Carey F. Coombs, 136.
Military general hospital in Egypt, On the work of a—E. H. Groves, 11.

Neuroses of the war, Some—Lieut.-Col. J. Michell Clarke, 49.
Norrington, Col. H. L., D.S.O.—Military honour conferred on, 100.

Obituary, 101, 201.
Orr, Ewing, Capt. H. J.—Military cross awarded to, 100.

Smith, F. Shingleton—Forensic medicine of blood-stains in the Tropics, 82.
Sphenoidal sinus explorations for meningococcal and other infections, Notes on the technique of—Major P. Watson-Williams, 21.
Sphenoidal sinus—Notes on the Operation for the drainage of the. By Major P. Watson-Williams, 157.
Surgery in France, Some aspects of British—Dr. James Swain, 128.
Surgery in Mesopotamia, Medicine and—Capt. Carey F. Coombs, 136.
Swain, Dr. James—Some aspects of British surgery in France, 128.

Teeth, The effect of general conditions on the quality of the—Major W. R. Ackland, 118.

Waldo, Dr. H.—Report on Dermatology, 178.
War, Some neuroses of the—Lieut.-Col. J. Michell Clarke, 49.
War, The reduction of disabilities from wounds in—W. Gordon, 1.
Watson-Williams, Major P.—Notes on the operation for drainage of the sphenoidal sinus, 157.
Watson-Williams, Major P.—Note on the technique of sphenoidal sinus exploration for meningococcal and other infections, 21.
White, Bart., Sir George, President of the Bristol Royal Infirmary—Death of, 194.
Wounds in war, The reduction of disabilities from—W. Gordon, 1.

Venereal diseases, The Local Government Board scheme for the treatment of, 188.

X-ray outfit, Ten months in France with a field service—Capt. W. Cotton, 144.

